# Glucose Rapidly Induces Different Forms of Excitatory Synaptic Plasticity in Hypothalamic POMC Neurons

**DOI:** 10.1371/journal.pone.0105080

**Published:** 2014-08-15

**Authors:** Jun Hu, Lin Jiang, Malcolm J. Low, Liangyou Rui

**Affiliations:** 1 Department of Molecular & Integrative Physiology, University of Michigan Medical School, Ann Arbor, Michigan, United States of America; 2 Department of Orthopedics, The First Affiliated Hospital of Nanjing Medical University, Nanjing, P. R. China; University of Texas Health Science Center at San Antonio, United States of America

## Abstract

Hypothalamic POMC neurons are required for glucose and energy homeostasis. POMC neurons have a wide synaptic connection with neurons both within and outside the hypothalamus, and their activity is controlled by a balance between excitatory and inhibitory synaptic inputs. Brain glucose-sensing plays an essential role in the maintenance of normal body weight and metabolism; however, the effect of glucose on synaptic transmission in POMC neurons is largely unknown. Here we identified three types of POMC neurons (EPSC(+), EPSC(−), and EPSC(+/−)) based on their glucose-regulated spontaneous excitatory postsynaptic currents (sEPSCs), using whole-cell patch-clamp recordings. Lowering extracellular glucose decreased the frequency of sEPSCs in EPSC(+) neurons, but increased it in EPSC(−) neurons. Unlike EPSC(+) and EPSC(−) neurons, EPSC(+/−) neurons displayed a bi-phasic sEPSC response to glucoprivation. In the first phase of glucoprivation, both the frequency and the amplitude of sEPSCs decreased, whereas in the second phase, they increased progressively to the levels above the baseline values. Accordingly, lowering glucose exerted a bi-phasic effect on spontaneous action potentials in EPSC(+/−) neurons. Glucoprivation decreased firing rates in the first phase, but increased them in the second phase. These data indicate that glucose induces distinct excitatory synaptic plasticity in different subpopulations of POMC neurons. This synaptic remodeling is likely to regulate the sensitivity of the melanocortin system to neuronal and hormonal signals.

## Introduction

Glucose is an essential metabolic fuel, and it also functions as a key metabolic signal to regulate metabolism and body weight [1]–[3]. Glucose-sensing neurons are located in several hypothalamic areas, including the lateral, ventromedial, and arcuate hypothalamus [1]. Glucose-excited neurons increase, whereas glucose-inhibited neurons decrease, their electrical activities as extracellular glucose concentrations increase from 0.1 to 5 mM [4], [5]. Brain glucose is normally maintained within a range of 0.7–2.5 mM, but it could further decrease to 0.2 mM in hypoglycemia or increase to 5 mM in hyperglycemia [1]. Four subpopulations of glucose-sensing neurons have been identified in the arcuate hypothalamus (ARC) based on the effect of glucose on their excitability [6]. The ARC is a key area for the maintenance of normal metabolism and body weight [7]. Hypothalamic glucose-sensing plays an important role in energy homeostasis and nutrient metabolism [8].

The ARC contains anorexigenic pro-opiomelanocortin (POMC) neurons which are chemically defined by coexpression of both POMC and cocaine-and amphetamine-regulated transcript neuropeptides [9]. Ablation of POMC neurons in the ARC results in hyperphagia and obesity in mice [10], [11]; in contrast, acute excitation of POMC neurons decreases food intake [12]. The hypothalamic melanocortin system plays an essential role in controlling nutrient metabolism and body weight in both rodents and humans [13].

The activity of POMC neurons is tightly regulated by nutrient, hormonal, and neuronal signals [13]. A subset of POMC neurons are glucose-excited neurons, and glucose directly excites these neurons by closing ATP-sensitive potassium (K_ATP_) channels [14], [15]. In contrast, insulin directly hyperpolarizes a subset of POMC neurons by activating K_ATP_ channels in a PI 3-kinase-dependent manner [16], [17]. POMC neurons express opioid μ receptors [15], [18], and dynorphin-A directly hyperpolarizes POMC neurons and depresses their electrical activity by activating G protein-gated inwardly rectifying potassium (GIRK) channels [15], [18], [19]. Unlike dynorphins, leptin and serotonin directly depolarize and excite hypothalamic POMC neurons by activating transient receptor potential C (TRPC) channels [14], [17], [20]–[24]. Additionally, nutrient, hormonal, and neuronal signals also regulate POMC neuron synaptic transmission. Inhibitory GABAergic inputs onto POMC neurons are higher in the fasted state [25]; in agreement, ghrelin, a fasting hormone, increases inhibitory inputs, but decreases excitatory glutamatergic inputs, onto POMC neurons [26]. In contrast, leptin increases excitatory inputs, but decreases GABAergic inputs, onto POMC neurons [25], [26]. Moreover, obesity is associated with abnormal synaptic transmission in hypothalamic POMC neurons [26], [27], raising the possibility that aberrant synaptic reorganization and remodeling may contribute to the pathogenesis of obesity and obesity-associated metabolic disease.

Glucose-excited neurons and glucose-inhibited neurons have been extensively characterized, but the effect of extracellular glucose on synaptic transmission in hypothalamic POMC neurons remains largely unknown. Excitatory glutamatergic terminals make synaptic connections with hypothalamic POMC neurons [28], and POMC neurons have more excitatory than inhibitory synapses under normal conditions [26]. We hypothesized that nutritional signals, including glucose, regulate excitatory glutamatergic inputs onto hypothalamic POMC neurons, thus modifying POMC neuronal activity. In this work, we performed whole-cell patch-clamp recordings of POMC neurons in acute hypothalamic slices. We described four types of POMC neurons in terms of their synaptic responses to lowering extracellular glucose (glucoprivation), and identified a novel type of POMC neurons whose excitatory synaptic inputs displayed a bi-phasic pattern in response to glucoprivation. These observations suggest that glucose-induced synaptic remodeling of hypothalamic POMC neurons may regulate the sensitivity of the melanocortin system to hormonal and neuronal signals, thus modulating the ability of this system to control body weight and metabolism.

## Results

### Glucose rapidly increases excitatory synaptic inputs onto hypothalamic POMC neurons

**Figure 1:**
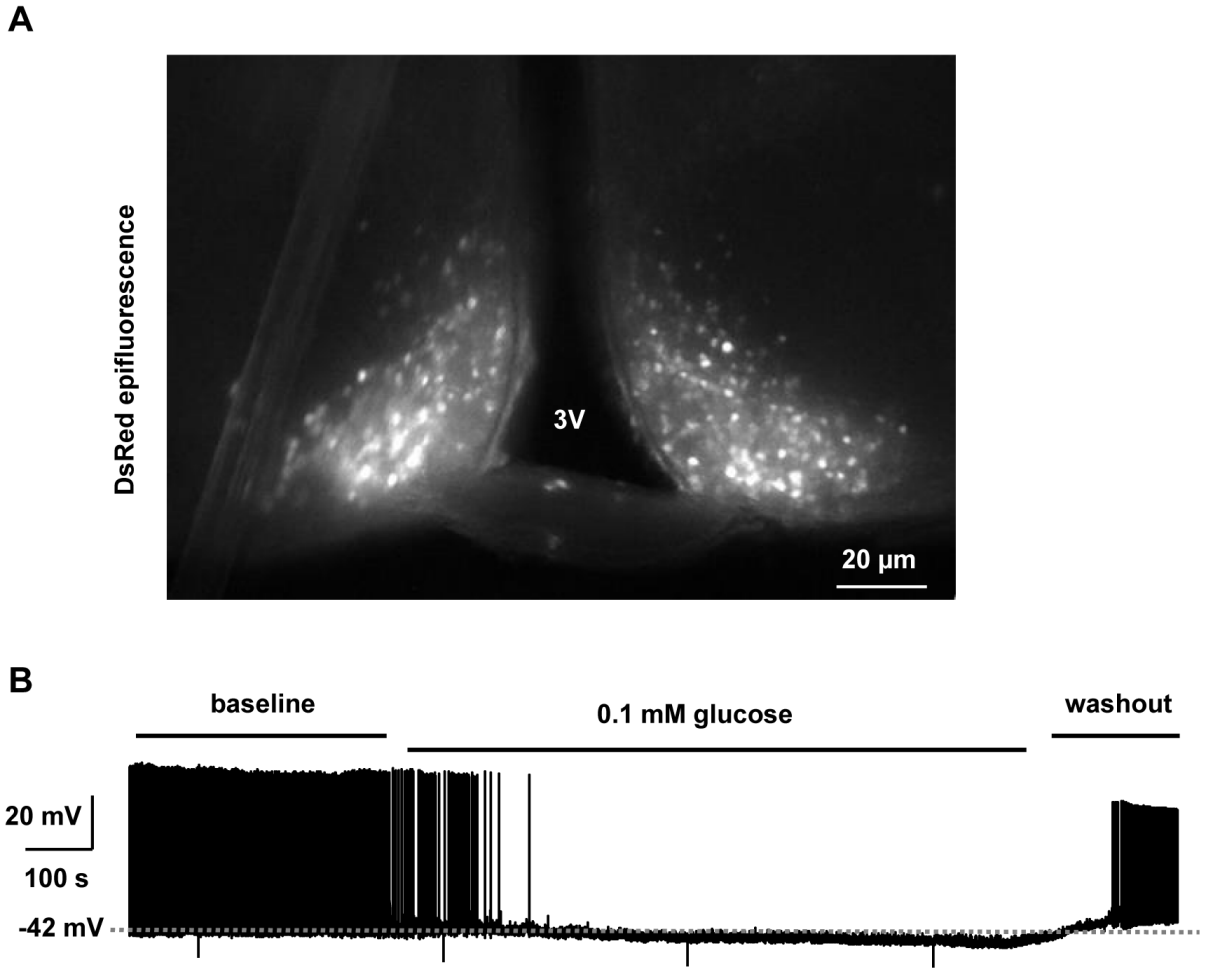
Glucose-excited POMC neurons in the ARC. (A) Hypothalamic sections containing the ARC were prepared from POMC DsRed mice (7 weeks), and POMC neurons were visualized using a fluorescent microscope. (B) Action potentials were recorded in the whole-cell current-clamp mode (injecting 10 pA depolarizing currents) in POMC neurons perfused sequentially with aCSF containing 5 mM glucose (baseline), 0.1 mM glucose, and 5 mM glucose (washout).

We used Discosoma red (DsRed) transgenic mice (7–9 weeks) to examine synaptic transmission in hypothalamic POMC neurons. These mice express DsRed specifically in POMC neurons, and DsRed epifluorescence was previously verified to be restricted to hypothalamic ACTH-positive neurons [29]. In agreement, DsRed was detected in the ARC where hypothalamic POMC neurons are exclusively located (Fig. 1A). We examined the electrical activity of POMC neurons in hypothalamic slices using whole-cell patch-clamp recordings. POMC neurons were identified by DsRed epifluorescence as previously described [29], [30]. A subset of POMC neurons spontaneously fired action potentials, and bath application of low-glucose (0.1 mM) hyperpolarized these neurons and markedly decreased their firing rates (Fig. 1B). Their electrical activities were recovered after washout of 0.1 mM glucose (Fig. 1B). These observations are consistent with previous reports that a subpopulation of hypothalamic POMC neurons consists of glucose-excited neurons [14], [15].

**Figure 2:**
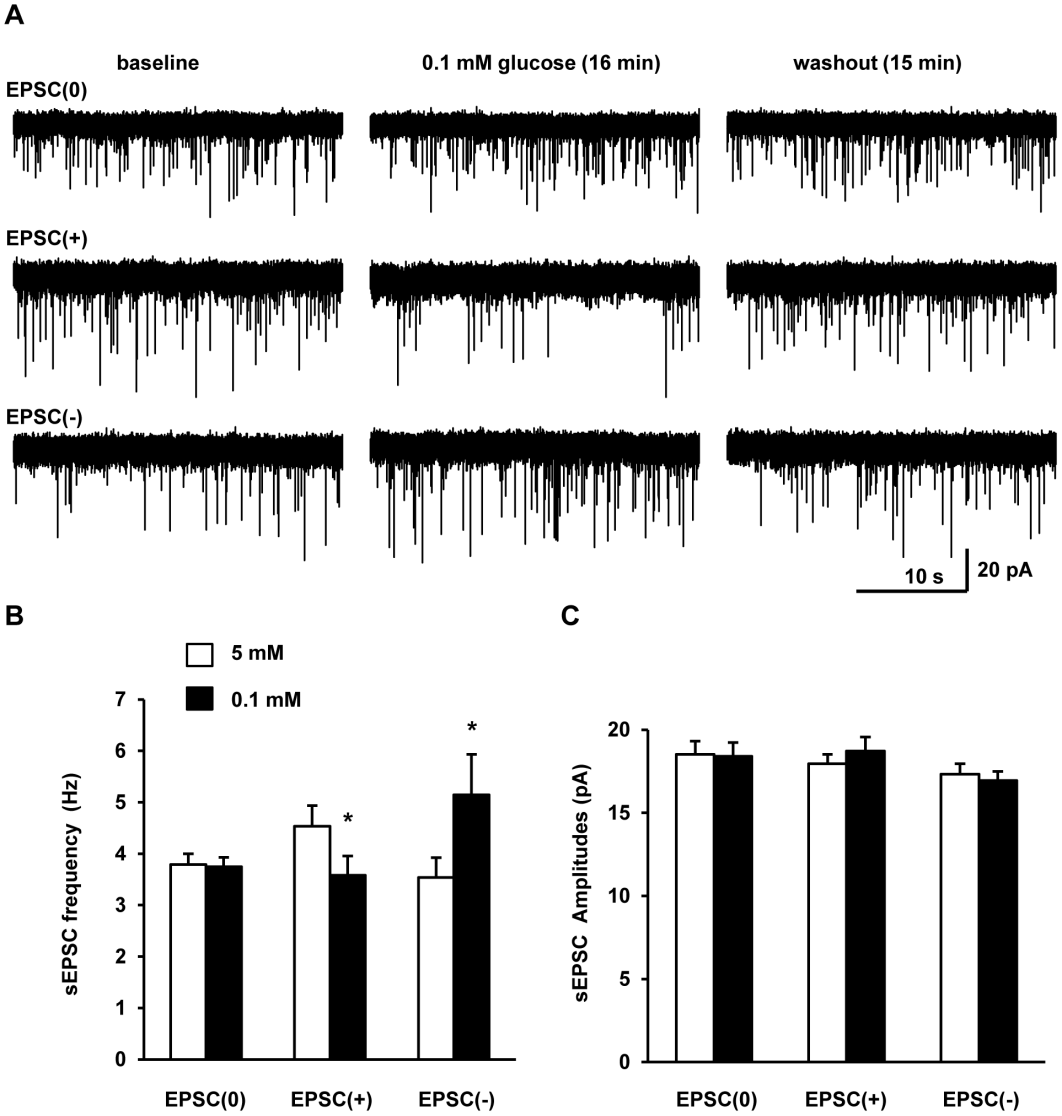
Three forms of glucose-regulated excitatory synaptic transmission in hypothalamic POMC neurons. Spontaneous EPSCs in POMC neurons were recorded in the whole-cell voltage-clamp mode at −60 mV holding potentials. GABAergic inhibitory synaptic transmission was blocked during the entire recording period by bath application of bicuculline methiodide (30 µM). After baseline recordings at 5 mM glucose for 15 min, sEPSCs were recorded for additional 30 min in brain slices perfused with aCSF containing 0.1 mM glucose. Glucose levels in aCSF were restored to 5 mM (washout), and sEPSCs were monitored for additional 20 min. (A) Representative traces of sEPSCs at baseline 5 mM glucose, 0.1 mM glucose (16 min after perfusion with 0.1 mM glucose), or washout (15 min after washout of 0.1 mM glucose). (B) Mean frequency of sEPSCs at baseline 5 mM glucose or 0.1 mM glucose (16 min after perfusion with 0.1 mM glucose). (C) Mean amplitude of sEPSCs. EPSC(0): n = 7, EPSC(+): n = 9, EPSC(−): n = 6. Data are presented as means ± SEM. *P*<0.05.

To examine the effect of glucose on glutamatergic synaptic transmission in POMC neurons, we measured spontaneous excitatory postsynaptic currents (sEPSCs) in the presence of bicuculline methiodide, a GABA_A_ receptor blocker. POMC neurons were held at −60 mV in a voltage-clamp mode. POMC neurons received strong excitatory synaptic inputs at baseline 5 mM glucose. Lowering extracellular glucose (0.1 mM) decreased the frequency of sEPSCs in a subset of POMC neurons (33.3%, 9 out of 27 neurons) (Fig. 2A–B). In contrast, the amplitude of sEPSCs was unchanged (Figs. 2A–B and 2C). We named this subpopulation EPSC(+). The reduction in sEPSC frequency was largely recovered after washout of 0.1 mM glucose (Fig. 2A).

**Figure 3:**
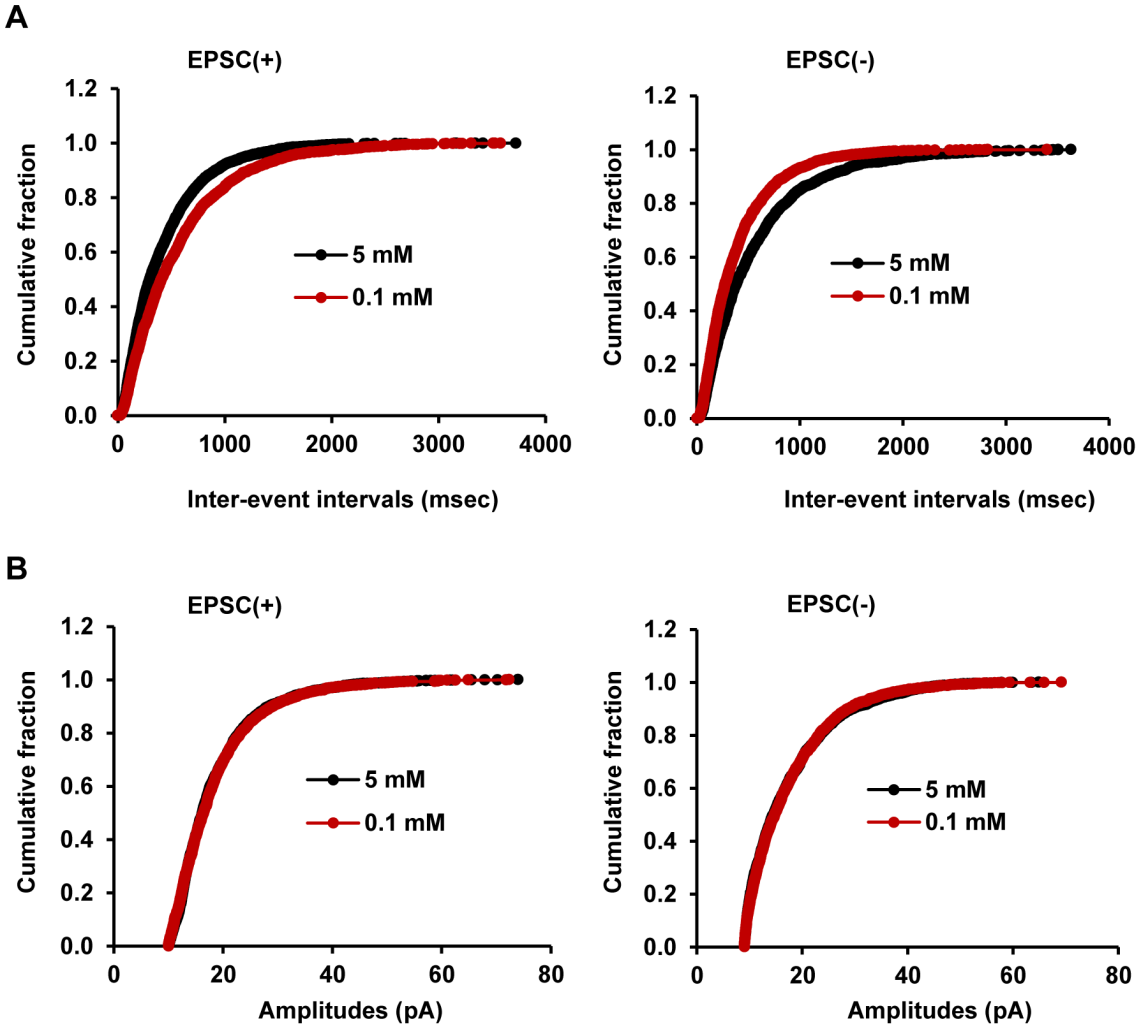
The opposite effect of glucoprivation on sEPSC distributions in EPSC(+) and EPSC(-) neurons. Spontaneous EPSCs were recorded in POMC neurons at baseline 5 mM glucose or 0.1 mM glucose (perfused with 0.1 mM glucose for 16 min) as described in Figure 2. (A) The inter-event distribution curves of sEPSCs. (B) The amplitude distribution curves of sEPSCs. EPSC(+): n = 9, EPSC(−): n = 6.

To further analyze the excitatory synaptic response of EPSC(+) neurons to glucose, we constructed the inter-event interval distribution curves and the amplitude distribution curves of sEPSCs. Lowering extracellular glucose caused a right shift of the inter-event interval distribution curves (Fig. 3A), further indicating that glucoprivation decreases sEPSC frequencies in EPSC(+) neurons. In contrast, the amplitude distribution curves were similar between 5 mM and 0.1 mM glucose (Fig. 3B). Together, these data indicate that lowering extracellular glucose decreases excitatory synaptic inputs onto the EPSC(+) subpopulation of hypothalamic POMC neurons, most likely by reducing presynaptic release of glutamate neurotransmitter.

Previous studies indicate that in acute ARC slices, tetrodotoxin (TTX) treatments do not change the frequency of EPSCs in POMC neurons [26], [30], [31], so we did not measure EPSCs in the presence of TTX.

### Hypothalamus POMC neurons are heterogeneous in terms of their glucose-regulated excitatory synaptic transmission

Leptin, insulin, and serotonin stimulate distinct, non-overlapping POMC neurons [21], [32], suggesting that hypothalamic POMC neurons are heterogeneous in their signaling properties. We identified a separate subpopulation of POMC neurons (22.2%, 6 out of 27 neurons), named EPSC(−), which displayed the opposite sEPSC response to glucoprivation. Lowering glucose increased the frequency of sEPSCs in EPSC(−) neurons (Fig. 2A–B), and caused a left shift of their inter-event interval distribution curves (Fig. 3A). Lowering glucose did not alter either the amplitude of sEPSCs (Figs. 2A and 2C) or the amplitude distribution curves in EPSC(−) neurons (Fig. 3B). These data indicate that glucoprivation increases the strength of excitatory synaptic transmission in the EPSC(−) subpopulation, presumably by enhancing presynaptic release of glutamate.

We also observed that in a subset of POMC neurons (25.9%, 7 out 27), designated as EPSC(0), glucoprivation did not alter either the frequency or the amplitude of their sEPSCs (Fig. 2).

### Identification of the bi-phasic EPSC(+/−) subpopulation of POMC neurons

**Figure 4:**
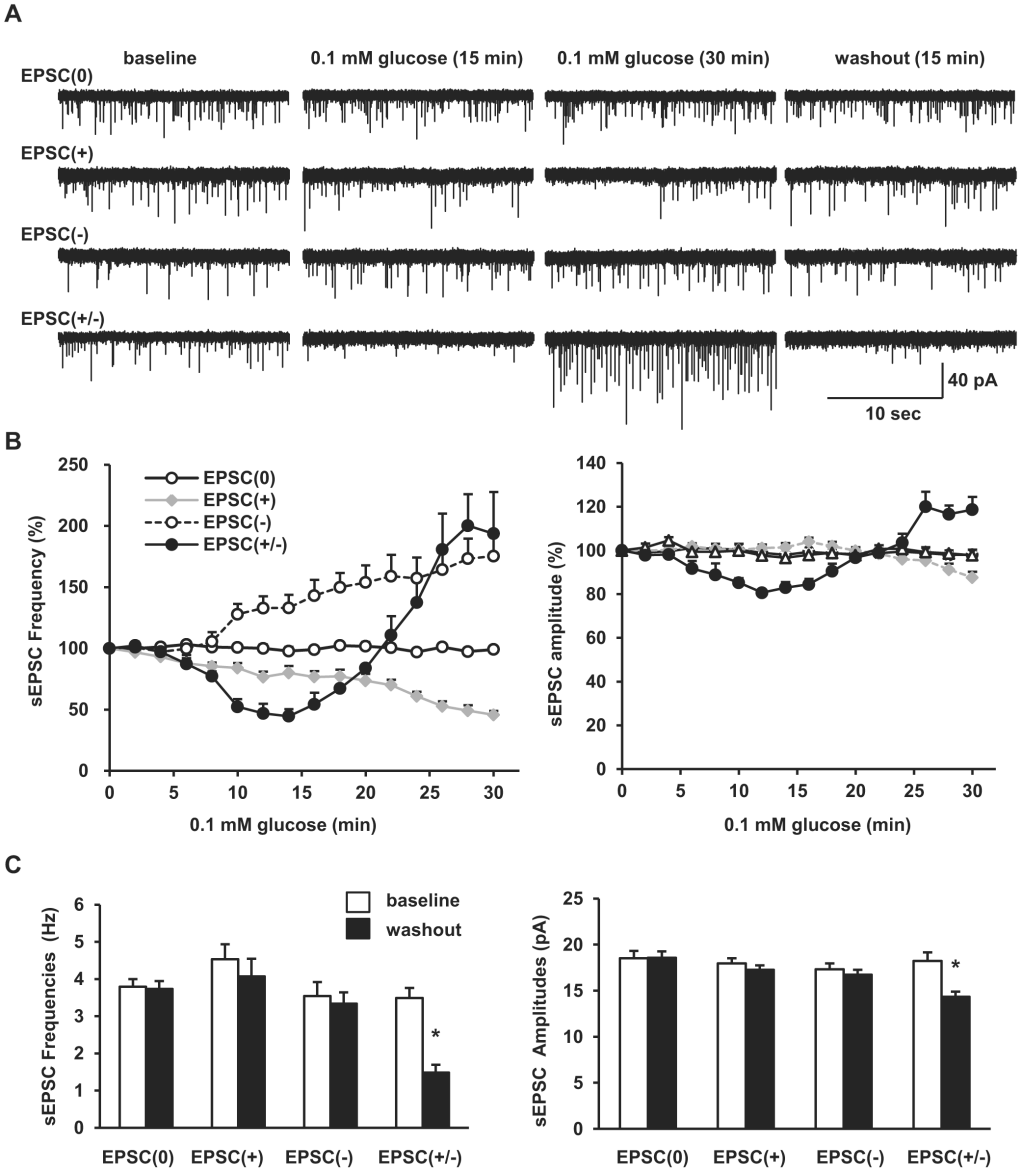
The dual effects of extracellular glucose on excitatory synaptic transmission in the EPSC(+/−) subpopulation. Spontaneous EPSCs were recorded in POMC neurons as described in Figure 2. (A) The traces of sEPSCs in four types of hypothalamic POMC neurons at baseline 5 mM glucose, 0.1 mM glucose for 15 min, 0.1 mM for 30 min, or washout of 0.1 mM glucose. (B) The mean frequency and the mean amplitude of sEPSCs were calculated every 2-min, normalized to baseline values, and plotted over time. (C) The mean frequency and the mean amplitude of sEPSCs at the baseline and washout conditions. EPSC(0): n = 7, EPSC(+): n = 9, EPSC(−): n = 6, EPSC(+/−): n = 5. Data are presented as means ± SEM. *P*<0.05.

We also identified the novel EPSC(+/−) subpopulation (18.5%, 5 out 27) that had a bi-phasic excitatory synaptic response to glucoprivation. Like EPSC(+) neurons, EPSC(+/−) neurons displayed glucoprivation-induced suppression of sEPSCs in the first phase (0–15 min after perfusion with 0.1 mM glucose), but the magnitude of sEPSC frequency change was larger in EPSC(+/−) neurons (Fig. 4A–B). In the second phase of glucoprivation (15–30 min after perfusion with 0.1 mM glucose), EPSC(+/−) neurons completely changed the direction of their synaptic response to glucoprivation, and the frequency of their sEPSCs increased progressively and was comparable to that in EPSC(−) neurons 25 min after 0.1 mM treatment (Fig. 4B). EPSC(+/−) neurons also displayed bi-phasic changes in the amplitude of their sEPSCs in response to glucoprivation. In the first phase, the amplitude of their sEPSCs decreased to the levels below the baseline; in the second phase, sEPSC amplitude progressively increased to the levels ∼20% higher than the baseline values (Figs. 4A–B). The frequency of sEPSCs in both EPSC(+) and EPSC(−) neurons was recovered approximately to their baseline levels 15 min after washout of 0.1 mM glucose; in contrast, both the frequency and amplitude of sEPSCs in EPSC(+/−) neurons were suppressed after washout (Fig. 4A and 4C). These data demonstrate that glucoprivation rapidly induces profound excitatory synaptic plasticity in EPSC(+/−) neurons, presumably by modulating both presynaptic release of glutamate and postsynaptic response to this neurotransmitter.

**Figure 5:**
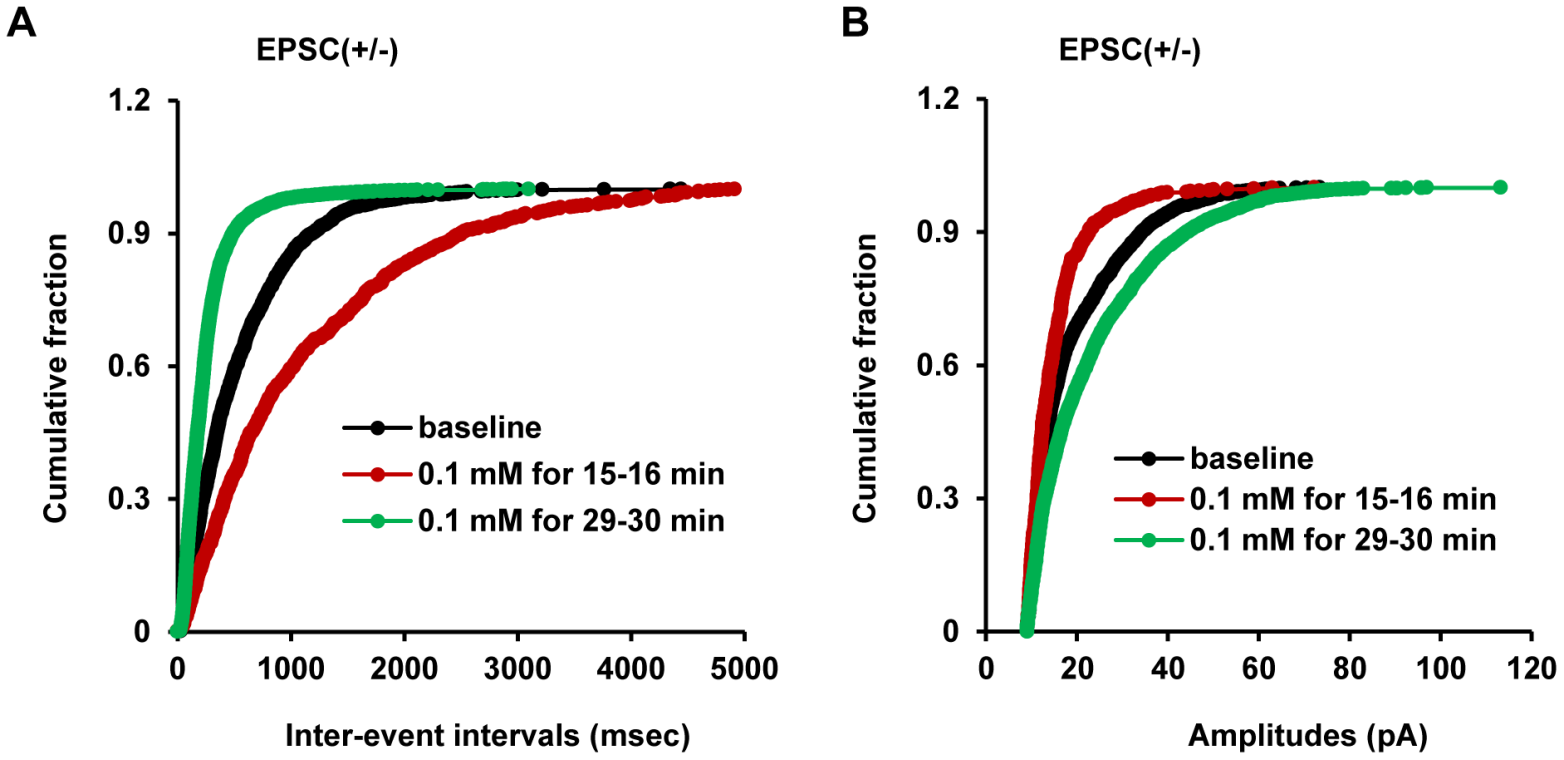
The bi-phasic effects of glucoprivation on sEPSC distributions in EPSC(+/−) neurons. (A) The inter-event interval distribution curves of sEPSCs in EPSC(+/−) neurons at baseline, 0.1 mM glucose for 15 min, or 0.1 mM glucose for 30 min. (B) The amplitude distribution curves of sEPSCs in EPSC(+/−) neurons at baseline, 0.1 mM glucose for 15 min, or 0.1 mM glucose for 30 min. EPSC(+/−): n = 5.

To further examine the effect of glucoprivation on excitatory synaptic transmission in EPSC(+/−) neurons, we analyzed the inter-event interval and the amplitude distribution curves of sEPSCs in the first phase (15 min after perfusion with 0.1 mM glucose) and the second phase (30 min after perfusion with 0.1 mM glucose). In the first phase, glucoprivation caused a right shift of the inter-event interval curves, which is similar to that in EPSC(+) neurons, whereas in the second phase, it caused a left shift, which resembles that in EPSC(−) neurons (Fig. 5A). These data further indicate that glucoprivation causes bi-phasic changes (decrease in the early phase and increase in the late phase) in excitatory glutamatergic synaptic transmission onto EPSC(+/−) neurons. Unlike that in EPSC(+) and EPSC(−) neurons, the amplitude distribution curves of sEPSCs were also shifted toward the left and the right during the first and the second phases of glucoprivation, respectively (Fig. 5B). These results suggest that in addition to modulating presynaptic release of glutamate, glucose also modulates postsynaptic glutamatergic transmission in EPSC(+/−) neurons. Together, these data suggest that glucoprivation induces profound reorganization and remodeling of excitatory synaptic transmission in the EPSC(+/−) subpopulation, resulting in a switch from the inhibitory to the excitatory states during prolonged hypothalamic glucopenia.

### Prolonged glucoprivation exerts bi-phasic effects on the electrical activity of a subset of hypothalamic POMC neurons

**Figure 6:**
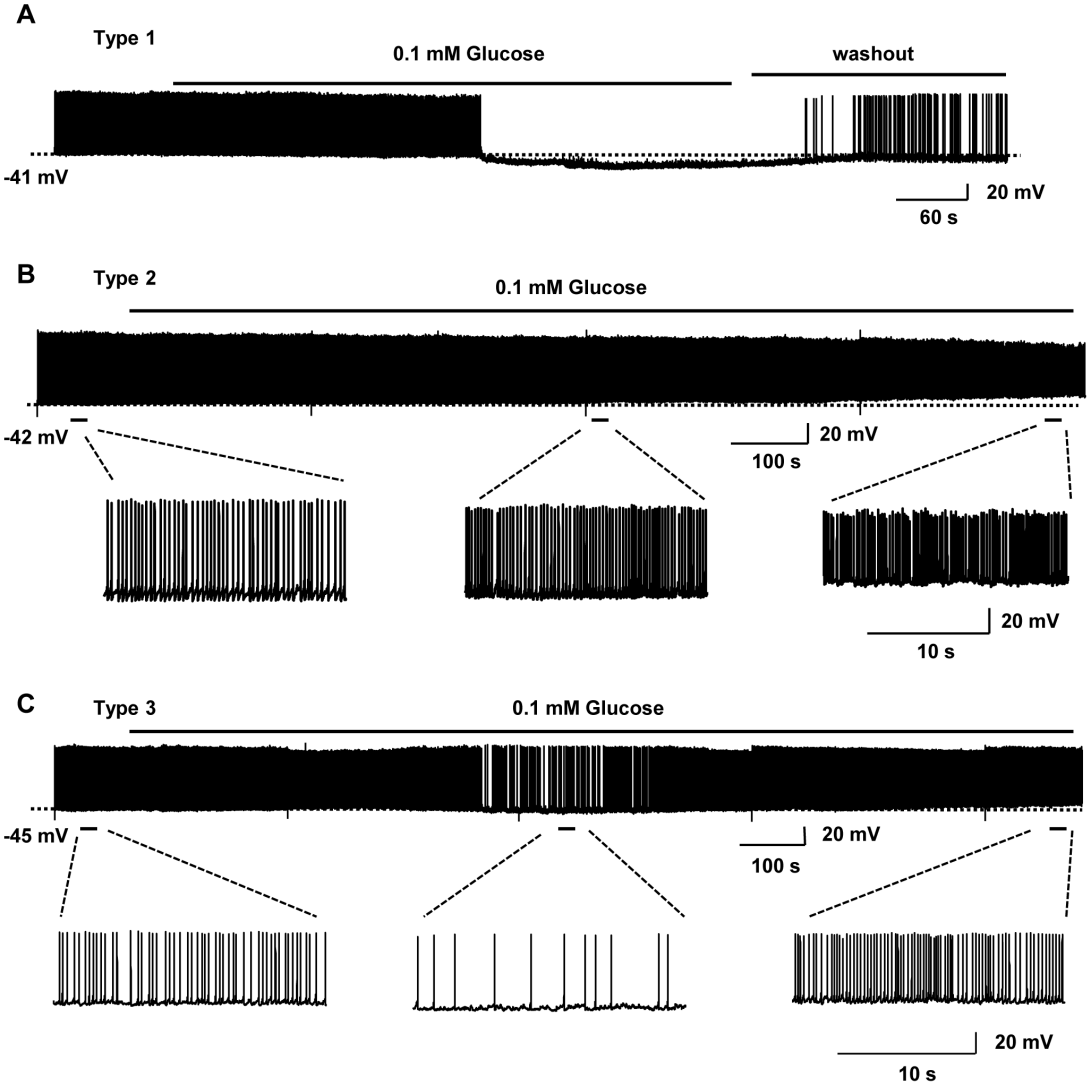
The effect of glucoprivation on the electrical activity of three types of POMC neurons. Action potentials were recorded in POMC neurons at different glucose concentrations in the whole-cell current-clamp mode (injecting 1–10 pA depolarizing currents). Representative traces of spontaneous firings at different glucose concentrations for various durations as indicated.

To determine whether altered synaptic transmission in POMC neurons affect their neuronal activities, we measured their action potentials in acute hypothalamic slices in whole-cell current-clamp recordings. We observed four types of glucose-sensitive POMC neurons. In type 1 neurons (27.6%, 8 out of 29), lowering glucose caused reversible hyperpolarization and suppression of action potentials during the entire glucoprivation period (Fig. 6A). In contrast, lowering glucose depolarized type 2 neurons and increased their firing rates (27.6%, 8 out of 29) (Fig. 6B). In type 3 neurons (13.8%, 4 out of 29), in the first phase, lowering glucose caused hyperpolarization and suppression of action potentials; by contrast, in the second phase, continuously lowering glucose induced depolarization and an increase in firing rates (Fig. 6C). Type 4 neurons (31.0%, 9 out of 29) did not electrically respond to glucoprivation. The pattern and kinetics of glucose response were very similar between action potentials in type 3 neurons and sEPSCs in EPSC(+/−) neurons (Figs. 4B and 6C), suggesting that type 3 neurons represent the EPSC(+/−) subpopulation. Type 1, 2 and 4 neurons may represent EPSC(+), EPSC(−), and EPSC(0) neurons, respectively.

## Discussion

In this study, we demonstrate that extracellular glucose has a profound effect on excitatory synaptic transmission in hypothalamic POMC neurons. We described three forms of glucose-regulated excitatory synaptic transmission in POMC neurons. Moreover, we have identified a novel action of glucose in inducing bi-phasic synaptic plasticity in EPSC(+/−) neurons.

We observed that hypothalamic POMC neurons are heterogeneous in terms of their synaptic response to extracellular glucose. Lowering extracellular glucose decreased the frequency of sEPSCs in EPSC(+) neurons, but it had the opposite effects in EPSC(−) neurons. Surprisingly, EPSC(+/−) neurons displayed the bi-phasic synaptic response to glucoprivation. In the first phase, lowering glucose decreased both the frequency and the amplitude of sEPSCs, whereas in the second phase, EPSC(+/−) neurons reversed their synaptic responses and progressively increased the frequency and the amplitude of their sEPSCs. In line with these findings, different subpopulations of POMC neurons have been reported based on their responses to leptin, insulin, or 5-HT stimulation [21], [32] and on their neurotransmitters (e.g. glutamate, GABA, or acetylcholine) [25], [29], [33]–[37]. These observations raise the possibility that different subpopulations of hypothalamic POMC neurons may regulate distinct aspects of energy and nutrient metabolism.

**Figure 7:**
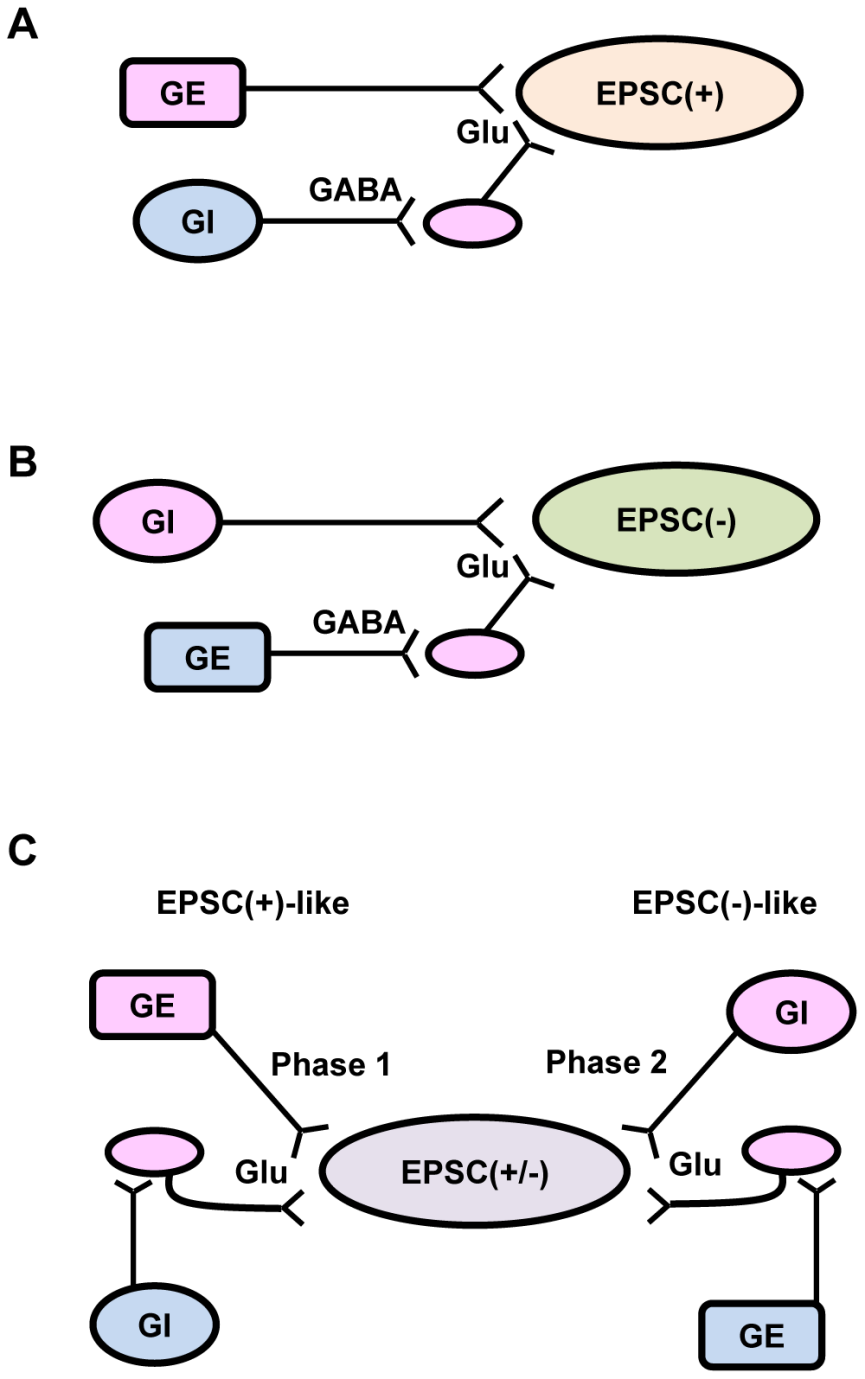
A model of glucose-regulated excitatory synaptic transmission in POMC neurons. (A) A EPSC(+) neuron was innervated directly by a glutamatergic, glucose-excited (GE) neuron (pink), and extracellular glucose stimulates the GE neuron to increase glutamatergic inputs onto the EPSC(+) neuron. Additionally, the EPSC(+) neuron may also be innervated indirectly by a GABAergic, glucose-inhibited (GI) neuron (blue) via a glutamatergic neuron (pink), and extracellular glucose causes disinhibition of the EPSC(+) neuron through this circuit. (B) A EPSC(−) neuron was innervated by a glutamatergic GI neuron, and glucose inhibits glutamatergic transmission in the EPSC(−) neuron. Additionally, the EPSC(−) neuron may also be innervated indirectly by a GABAergic GE neuron via a glutamatergic neuron. Glucose inhibits this glutamatergic neuron by exciting the inhibitory GE neuron, thus decreasing the frequency of sEPSCs in the EPSC(−) neuron. (C) A EPSC(+/−) neuron is regulated by EPSC(+)-like connections during the first phase of glucoprivation (left) and by EPSC(−)-like connections during the second phase (right).

The source of presynaptic glutamatergic projections to POMC neurons is largely unknown. Glucose-sensing neurons are distributed in multiple brain areas, including the ARC, ventromedial hypothalamus (VMH), paraventricular hypothalamus, lateral hypothalamus, amygdala, and the brainstem [1], [38]. The VMH has glutamatergic projections to POMC neurons, providing a source of excitatory inputs [39]. The molecular and neuronal mechanisms of glucose action on different subpopulations of POMC neurons are not fully understood. Glucose directly excites glucose-excited (GE) neurons by closing K_ATP_ channels [14], [40], and it depresses glucose-inhibited (GI) neurons through chloride channels [5], [6]. 5′-AMP-activated protein kinase (AMPK) is also involved in glucose sensing [41]–[43]. We propose a model to explain the heterogeneity of glucose-induced synaptic remodeling in POMC neurons (Fig. 7). GE neurons provide directly glutamatergic projections to EPSC(+) neurons (Fig. 7A). Additionally, GI neurons may provide GABAergic synaptic inputs onto a glutamatergic neuron that in turn innervates EPSC(+), so glucose promotes disinhibition of EPSC(+) neurons through this circuit. In contrast, EPSC(−) neurons may connect to GE and GI neurons in the opposite manner (Fig. 7B). EPSC(+/−) neurons may have EPSC(+)-like and EPSC(−)-like dual innervations (Fig. 7C). In the first phase of glucoprivation, the EPSC(+)-like mechanism is predominant, whereas in the second phase, the EPSC(−)-like mechanism becomes dominant. Glucoprivation alters both the frequency and the amplitude of sEPSCs in EPSC(+/−) neurons, suggesting that lowering extracellular glucose induces both presynaptic and postsynaptic plasticity in this subpopulation. However, our data do not exclude the possibility that additional neural mechanisms may also be involved in glucose-sensing processes in EPSC(+), EPSC(−) and EPSC(+/−) neurons. Glucose levels may affect O-GlcNAc modifications of key proteins which regulate presynaptic and/or postsynaptic transmissions, thus contributing the observed results.

In conclusion, we have described multiple forms of glucoprivation-induced excitatory synaptic plasticity in POMC neurons. The physiological significance of the heterogeneity of glucose-regulated synaptic plasticity in POMC neurons is currently unknown. Perhaps, it may differentially modulate the activity of different melanocortin circuits which regulate different aspects of energy and nutrient metabolism. Moreover, glucose-regulated synaptic plasticity of the hypothalamic melanocortin circuits may provide an important neural substrate which governs energy and nutrient metabolism by integrating hormonal and neuronal signals.

## Materials and Methods

### Animals

Animal experiments were conducted following the protocols approved by the University Committee on the Use and Care of Animals (UCUCA) at the University of Michigan Medical School. Discosoma red (DsRed) transgenic mice (in C57BL/6 background) were described previously [29]. The expression of DsRed is under the control of the mouse *POMC* promoter (from −13.3/−6.8 and −2.1+3.2 kb relative to the transcript start site). POMC neuron-specific expression of DsRed has been verified by colocalization of DsRed fluorophore and immunoreactivity for ACTH in the hypothalamus [29]. Mice were fed a normal chow diet and tap water *ad libitum* and housed on a 12-h light-dark cycle in the Unit for Laboratory Animal Medicine at the University of Michigan (ULAM).

### Preparation of hypothalamic slices

Male mice (7–9 weeks) were sacrificed by decapitation, and brains were rapidly removed and immersed in ice-cold, oxygenated (95% O_2_, 5% CO_2_) section solution (in mM): 220 sucrose, 10 D-glucose, 5 KCl, 3 MgCl_2_, 1 CaCl_2_, 1.2 NaH_2_PO_4_, 26 NaHCO_3_, pH 7.35. Coronal brain slices (300 µm), containing the ARC, were cut in ice-cold, oxygenated section solution using a Leica VT1200 vibratome (Leica Biosystems Nussloch GmbH, Nussloch, Germany). Two-three slices were prepared from each mouse. Prior to electrical recordings, brain slices were maintained at room temperature for at least 1 h in oxygenated (95% O_2_, 5% CO_2_) artificial cerebrospinal fluid (aCSF) (in mM): 120 NaCl, 5 KCl, 2 CaCl_2_, 1 MgCl_2_, 26 NaHCO_3_, 1.2 NaH_2_PO_4_, 5 D-glucose, adjusted to pH 7.4 with NaOH.

### Electrophysiology

Brain slices were transferred into a recording chamber (RC-22C, Warner Instruments, Hamden, CT, USA) which was continuously perfused with aCSF (saturated with 95% and 5% CO2) at 1–2 ml per min. aCSF was heated (at 33°C) using a SH-27B solution heater and a TC-324B perfusion heater controller (Warner Instruments). The recording chamber was mounted on a BX51 WI upright microscope (Olympus, Tokyo, Japan) equipped with a 40× water immersion objective (Olympus), an infrared-differential interference contrast (IR-DIC) optical system, a monochrome CCD camera (Olympus), and a monitor. Recording pipettes were pulled from borosilicate glass capillary tubes (1.5–1.8×100 MM, Kimble Chase) using a P1000 micropipetter puller (Sutter Instrument, Novato, CA, USA). They were filled with an internal solution (in mM): 120 K-gluconate, 10 KCl, 1 EGTA, 1 MgCl_2_, 0.5 CaCl_2_, 10 HEPES, 2 MgATP, 0.5 Na_2_GTP, adjusted to pH 7.3 with KOH, and had a tip resistance of 2.5–4.5 mΩ. Recording pipettes were connected via an Ag-AgCl wire to the headstage of an EPC-10/2 dual patch-clamp amplifier (HEKA Instruments Bellmore, NY, USA). The reference electrode had a silver-silver chloride pellet and was immersed in the bath solution. The amplifier filter was set at 5 kHz, and pipette and cell capacitance were compensated for using PatchMaster 2.20 software (HEKA Elektronik, Lambrecht/Pfalz, Germany). POMC neurons were identified by DsRed epifluorescence and patched under IR-DIC optics. Neurons with a series resistance larger than 20 mΩ were excluded from statistics. POMC neurons were held at −60 mV and sampled at 20 kHz for the entire recording period. Resting membrane potentials were determined from slow time-scale recordings. Spontaneous EPSCs were recorded in a whole-cell voltage-clamp mode at −60 mV holding potential with bath application of GABA_A_ receptor blocker bicuculline methiodide (30 µM, Sigma).

### Data collection and analysis

Data were acquired and analyzed using PatchMaster 2.20 running on a Windows XP operating system. Traces were processed using Igor Pro 4.07 (Wavemetrics, Lake Oswego, OR, USA). Spontaneous excitatory postsynaptic currents (sEPSCs) were analyzed using MiniAnalysis 6.03 (Synaptosoft Inc., Decatur, GA, USA). The inter-event interval curves and the frequency vs amplitude curves of sEPSCs were constructed using MiniAnalysis. Mean frequency and mean amplitude (in every 2-min) were calculated using MiniAnalysis.

### Statistical analysis

Data in each group were obtained from 3–6 mice and presented as means ± SEM. Paired t-tests were performed to compare responses of individual neurons to changes in extracellular glucose concentrations within each category of POMC neurons. P<0.05 was considered statistically significant.
